# Neferine Promotes GLUT4 Expression and Fusion With the Plasma Membrane to Induce Glucose Uptake in L6 Cells

**DOI:** 10.3389/fphar.2019.00999

**Published:** 2019-09-04

**Authors:** Ping Zhao, Di Tian, Guanjun Song, Qian Ming, Jia Liu, Jinhua Shen, Qing-Hua Liu, Xinzhou Yang

**Affiliations:** ^1^Institute for Medical Biology & Hubei Provincial Key Laboratory for Protection and Application of Special Plants in the Wuling Area of China, College of Life Sciences, South-Central University for Nationalities, Wuhan, China; ^2^National Demonstration Center for Experimental Ethnopharmacology Education, South-Central University for Nationalities, Wuhan, China; ^3^School of Pharmaceutical Sciences, South-Central University for Nationalities, Wuhan, China; ^4^Hubei Medical Biology International Science and Technology Cooperation Base, Wuhan, China

**Keywords:** neferine, glucose transporter 4, Ca^2+^, L6 cells, type 2 diabetes

## Abstract

Glucose transporter 4 (GLUT4) is involved in regulating glucose uptake in striated muscle, liver, and adipose tissue. Neferine is a dibenzyl isoquinoline alkaloid derived from dietary lotus seeds and has multiple pharmacological effects. Therefore, this study investigated neferine’s role in glucose translocation to cell surface, glucose uptake, and GLUT4 expression. In our study, neferine upregulated GLUT4 expression, induced GLUT4 plasma membrane fusion, increased intracellular Ca^2+^, promoted glucose uptake, and alleviated insulin resistance in L6 cells. Furthermore, neferine significantly activated phosphorylation of AMP-activated protein kinase (AMPK) and protein kinase C (PKC). AMPK and PKC inhibitors blocked neferine-induced GLUT4 expression and increased intracellular Ca^2+^. While neferine-induced GLUT4 expression and intracellular Ca^2+^ were inhibited by G protein and PLC inhibitors, only intracellular Ca^2+^ was inhibited by inositol trisphosphate receptor (IP_3_R) inhibitors. Thus, neferine promoted GLUT4 expression *via* the G protein-PLC-PKC and AMPK pathways, inducing GLUT4 plasma membrane fusion and subsequent glucose uptake and increasing intracellular Ca^2+^ through the G protein-PLC-IP_3_-IP_3_R pathway. Treatment with 0 mM extracellular Ca^2+^ + Ca^2+^ chelator did not inhibit neferine-induced GLUT4 expression but blocked neferine-induced GLUT4 plasma membrane fusion and glucose uptake, suggesting the latter two are Ca^2+^-dependent. Therefore, we conclude that neferine is a potential treatment for type 2 diabetes.

## Introduction

Glucose is an important energy source for muscle contraction. Normal glucose metabolism is vital for maintaining good health, and abnormal glucose metabolism may lead to serious health problems such as diabetes. As previous studies have shown, an increase in glucose uptake is caused by a change in the distribution of glucose transporters from intracellular storage vesicles to the plasma membrane in fat and skeletal muscle cells (Vincent et al., 2005; Pessin and Saltiel, 2000; Leto and Saltiel, 2012). Glucose transporter 4 (GLUT4) is the main transporter of glucose in muscle and fat cells and functions to control cellular glucose metabolism and whole-body energy homeostasis, which are both strongly linked to type 2 diabetes (Kubota et al., 2011; Huang et al., 2016; Gao et al., 2017). The selective downregulation of GLUT4 in adipose and muscle tissues can cause insulin resistance which increases the risk of developing diabetes (Abel et al., 2001; Leney and Tavare, 2009). Some studies have found that external stimulation increases glucose transport within organisms. For example, higher glucose transport during exercise mainly occurs due to the higher amounts of glucose transport protein GLUT4 on the surface membrane, specifically the sarcolemma and transverse tubules (Rose and Richter, 2005). In addition, drug stimulation, such as that by chloroquine or ergosterol from *Pleurotus ostreatus* (Zhou et al., 2016), also promotes GLUT4 expression and transport, thus promoting glucose uptake in L6 cells. Therefore, based on previous studies of GLUT4 expression and fusion with the plasma membrane, we believe it would be beneficial to further study the pathogenesis of type 2 diabetes to discover potential relevant therapeutic drugs.

The GLUT4 storage vesicle (GSV) is a transportable vesicle carrier found in fat and skeletal muscle cells. It comprises three main cargo proteins: GLUT4, insulin responsive aminopeptidase (IRAP), and sortilin. GSVs transfer GLUT4 to the plasma membrane under the stimulation of insulin, thus inducing intracellular glucose uptake. Other research has shown that this process is regulated by activating phosphatidylinositol 3-kinase (PI3K)/threonine kinase Akt, AMP-activated protein kinase (AMPK), and atypical protein kinase C (aPKC) (Grillo et al., 2009). Another study showed that insulin-receptor/insulin-receptor substrate-1 (IRS-1) is also involved in this process (Saltiel and Kahn, 2001). Previous studies revealed that both the increase in cytosolic Ca^2+^ concentration and the activation of AMPK during muscle contraction are involved in mediating the stimulation of glucose transport by contractile activity (Wright et al., 2004; Wright, 2007). These studies provide several concepts for studying the mechanism and potential treatments of type 2 diabetes.

In the past, natural products have played a significant role in the discovery of drugs for the treatment of diseases such as diabetes (Yao et al., 2008; Huang et al., 2010; Huang et al., 2018; Xiong et al., 2018; Zheng et al., 2018). In China, lotus seeds are extensively consumed as a food product, and the plant is also utilized as a source of traditional medicine. The extracts from lotus leaves, rhizomes, and seeds have been shown to possess multiple health benefits and contain a diverse amount of secondary metabolites (Bhat et al., 2009). Neferine, the focus of this study, is a dibenzyl isoquinoline alkaloid derived from lotus seeds (Zhao et al., 2014; Deng et al., 2016). Previous studies have shown that neferine has several pharmacological actions. For example, neferine significantly reduces the area of platelet adhesion collagen in mice and inhibits *in vitro* thrombosis (Zhou et al., 2013). Regarding isoproterenol-induced myocardial infarction, neferine can be used as a potent cardioprotective agent against isoproterenol-induced oxidative stress (Lalitha et al., 2013). In addition, in some animal experiments, neferine showed both anti-arrhythmic and hypotensive effects (Li et al., 2013). In another study conducted in insulin-resistant rats, neferine significantly reduced fasting blood glucose, insulin, triglycerides, tumor necrosis factor-α, and increased insulin sensitivity (Pan et al., 2009). In our study, we confirmed that neferine promotes GLUT4 expression *via* the G protein-PLC-PKC and AMPK pathways, thus promoting GLUT4 fusion with the plasma membrane and glucose uptake. We also confirmed that intracellular Ca^2+^ concentration was increased through the G protein-PLC-IP_3_-IP_3_R pathway, and that neferine-induced GLUT4 fusion with the plasma membrane and glucose uptake were both Ca^2+^-dependent. Together, these studies provide strong evidence that neferine could be developed as a drug for type 2 diabetes.

## Materials and Methods

### Chemicals and Reagents

**Figure 1:**
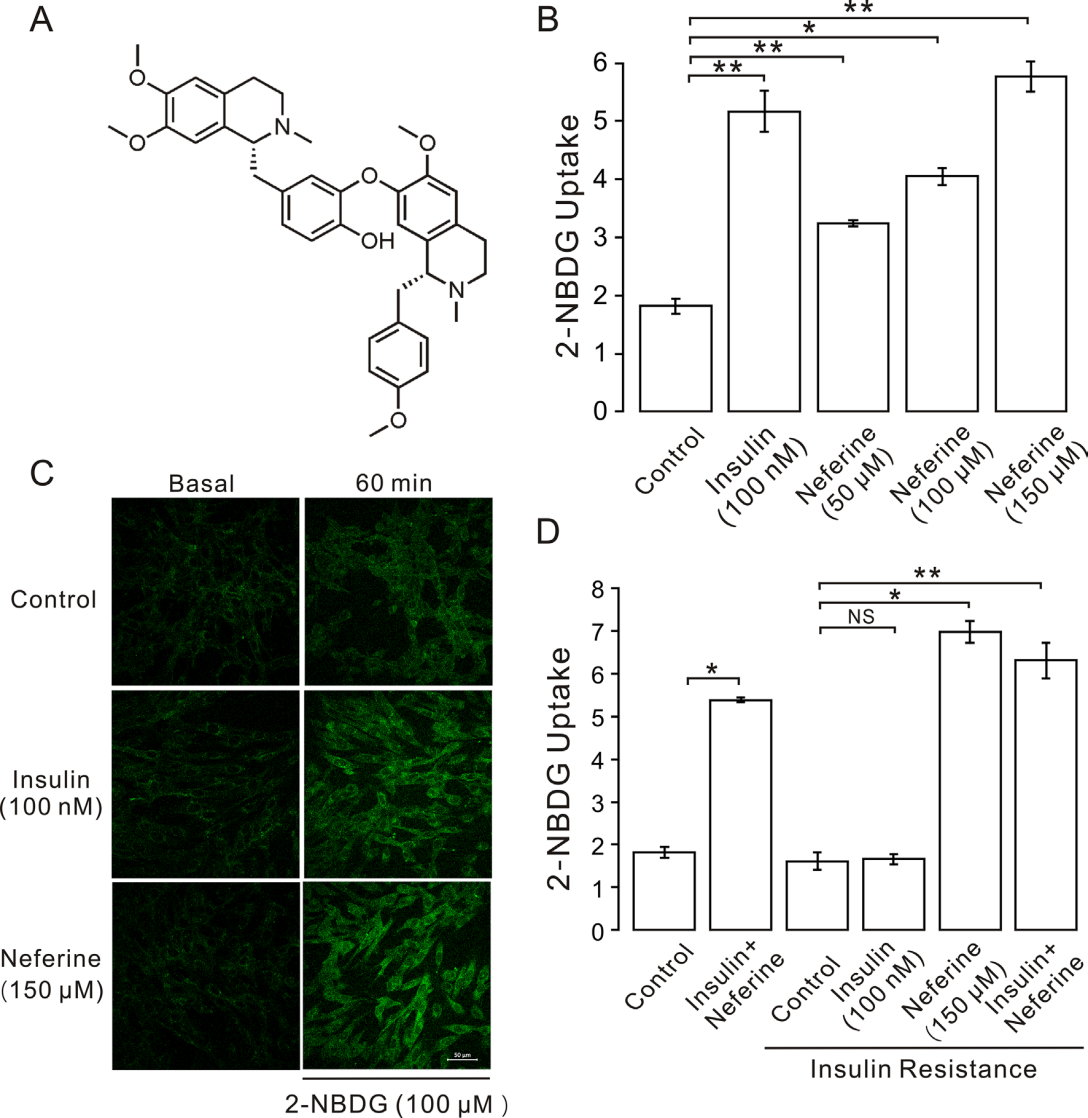
Neferine promotes glucose uptake in L6 cells. **(A)** Chemical structure of neferine. **(B)** Glucose uptake in L6 cells detected using 2-NBDG assay. Neferine and insulin both show increased glucose uptake. Neferine-induced glucose uptake is dose-dependent. **(C)** Images of 100 nm insulin and 150 μM neferine enhancing 2-NBDG uptake. Scale bar = 50 μm. **(D)** 150 μM neferine increased glucose uptake in insulin-resistant cells. NS *P* > 0.05; *P < 0.05; **P < 0.01.

Neferine was purchased from Shanghai Yuanye Bio-Technology Company (Shanghai, China). HPLC ≥98%. The chemical structure of neferine was shown in **Figure 1A**. The GLUT4 antibody, Akt antibody, phospho-Akt (Ser473) antibody, phospho-AMPK antibody, AMPK antibody, and phospho-PKC (pan) (Thr410) antibody were purchased from Cell Signaling Technology (Beverly, MA). The anti-c-myc mouse monoclonal antibody and FITC antibody were both purchased from TransGen Biotech (Beijing, China). Gö6976 and Gö6983 (PKC inhibitors) were purchased from EMD Millipore (Billerica, MA). Compound C (AMP-kinase inhibitor) was purchased from Calbiochem (San Diego, CA). Wortmannin (PI3K inhibitor) was purchased from Selleckchem (Houston, TX). BAPTA-AM (Ca^2+^ chelator), U73122 (PLC inhibitor), ryanodine (RyR receptor), and 2-APB (IP_3_R inhibitor) were purchased from Sigma–Aldrich (St. Louis, MO). U73343 (inactive analogue of U73122) was purchased from APExBIO (Houston, USA). Pertussis toxin (PTX) (G_α_ protein inhibitor) and gallein (G_β/γ_ protein inhibitor) were both purchased from Tocris Bioscience (Bristol, UK). Fluo-4 AM was purchased from Invitrogen (Carlsbad, CA). The 2 mM Ca^2+^ in physiological saline solution (PSS) contained the following (in mM): 135 NaCl, 5 KCl, 1 MgCl_2_, 2 CaCl_2_, 10 HEPES, and 10 glucose (pH = 7.4 adjusted with NaOH). The 0 mM Ca^2+^ PSS contained the following (in mM): 135 NaCl, 5 KCl, 1 MgCl_2_, 0.5 EGTA, 10 HEPES, and 10 glucose (pH = 7.4). Phosphate-buffered saline (PBS) contained the following (in mM): 137 NaCl, 10 Na_2_HPO_4_, 2.7 KCl, and 2 KH_2_PO_4_ (pH = 7.4). HES buffer contained the following (in mM): 250 sucrose, 20 HEPES, and 10 EDTA (pH = 7.4).

### Culture of L6 Cells, Development of Insulin-Resistant Cells, and Glucose Uptake Assays

L6 cells are provided by Professor Pingsheng Liu (Institute of Biophysics, the Chinese Academy of Science). L6 cell culture medium was prepared with 10% serum, 1% antibiotics (100 U/ml penicillin and 100 μg/ml streptomycin) and 89% minimum essential medium alpha modification (α-MEM, product code is 12571063; Gibco, Grand Island, NY). L6 myoblasts were cultured at 37°C in a 5% CO_2_ incubator. For differentiation, the cells were maintained in differentiation media containing 2% FBS for 5–7 days to acquire myotubes. In this study, glucose uptake levels of L6 myotubes were measured using the 2-NBDG Assay Kit (Cayman Chemical, Ann Arbor, MI). Firstly, L6 myoblasts were seeded on pre-treated glass coverslips, with each coverslip containing 1-ml culture medium. Then, L6 myoblasts are differentiated to L6 myotubes. To obtain insulin-resistant cells induced by high insulin concentration, cells were cultured with 1 μM insulin for 24 h while control cells were incubated with normal α-MEM medium. Before starting experiments, the cells were washed with PBS buffer and alpha MEM starved for at least 1 h. Initial fluorescent intensity in cells on glass coverslips was first detected by laser-scanning confocal microscopy (LSM 700; Carl Zeiss, Jena, Germany). Then, the buffer was replaced with 100 µl of sodium buffer containing insulin and neferine with different concentration for a 10-min incubation, to which was added 2-NBDG (100 μM final concentration) in 100 μl of sodium buffer as positive control and experimental groups, respectively. For the control, only 100 μl sodium buffer incubate 10 min and 100 μM 2-NBDG incubate 60 min. After being incubated at 37°C for 60 min, the buffer was discarded, and the cells were rinsed three times in PBS buffer, and the fluorescence in cells was acquired by laser-scanning confocal microscopy. Excitation = 490 nm; emission = 525 nm in the FITC range (Dhanya et al., 2017). The ratio of final fluorescent intensity to initial fluorescent intensity as the ordinate can reflect 2-NBDG uptake directly.

### Construction of myc-GLUT4-mOrange Plasmid and Cell Line

First, we inserted GLUT4-mOrange into a GV348 vector, and then the human c-myc epitope (14 amino acids) was inserted into the first ectodomain of GLUT4. Previous research showed that the epitope does not affect GLUT4 activity (Kanai et al., 1993). Next, lentivirus was prepared by transfecting the GV348-myc-GLUT4-mOrange vector. The supernatant of the transfected cells was collected after 48 h and centrifuged at 4°C, 4,000 g for 10 min. After removing the cell fragments, the supernatant was filtered into a 40-ml overspeed centrifuge tube using a 0.45-μm filter and was centrifuged at 4°C, 25,000 rpm for 2 h. Finally, the precipitate was suspended. The lentivirus was purified by concentration, and the corresponding virus quality was then measured. After the quality was confirmed, L6 cells were transfected with the lentivirus. The multiplicity of infection (MOI) = 100. First, cells were cultured in a six-well plate. Next, the diluted lentivirus (20 μl), the infectious reagent P (40 μl), and culture medium (760 μl) were gently mixed together and then added to the plate. After 24 h, intracellular fluorescence was observed. Cells were selected by growth in puromycin containing media, and finally, L6 cell lines containing myc-GLUT4-mOrange were obtained.

### GLUT4 Expression Assays

Myc-GLUT4-mOrange-L6 myoblasts were cultured on glass coverslips overnight, and then, L6 myoblasts are differentiated to L6 myotubes; the cells were starved in a PSS solution for 2 h. Subsequently, laser-scanning confocal microscopy (LSM 700; Carl Zeiss, Jena, Germany) was used to measure mOrange fluorescence at an excitation wavelength of 555 nm. After cells were treated with 150 μM neferine, images were taken every 5 min over a period of 30 min. The fluorescence intensity of mOrange was then analyzed using the Zen 2010 Software (Carl Zeiss, Jena, Germany).

### Intracellular Ca^2+^ Assays

L6 cells were cultured on glass coverslips overnight, and then, the cells were starved in a PSS solution for 2 h. Next, the cells were treated with 2 mM Fluo4-am in PSS and incubated at room temperature for 20 min. Then, cells were detected using an LSM 700 laser-scanning confocal microscope, with Fluo-4 AM fluorescence measured using a 488-nm filter. Images were taken 10 seconds before neferine treatment and every 5 min after treatment. Changes in cytoplasmic Fluo-4 fluorescence were analyzed and recorded using Zen 2010 software.

### GLUT4 Fusion With the Plasma Membrane

Myc-GLUT4-mOrange cells were cultured in six-well plates and grown on coverslips. After 2 h of starvation treatment, the cells were incubated in the presence of neferine or insulin for 30 min. Then, cells were then fixed with 3% polyformaldehyde for 30 min. After blocking with 2% bovine serum albumin (BSA) in PBS for 30 min at room temperature, the cells were incubated with anti-myc mouse monoclonal antibody for 1 h at room temperature. Then, the cells were washed three times with 2% BSA in PBS and incubated with goat anti-mouse-FITC. After being washed three times with 2% BSA in PBS, the coverslips were turned over and placed on a glass slide. Finally, mOrange red fluorescence and FITC green fluorescence were observed using a laser confocal microscope. GLUT4 externalization was quantitated by determination of the percentage of GLUT4-mOrange-positive cells that exhibited FITC fluorescence at the cell surface. Data are means ± SEM of values from three separate experiments, with 180 to 250 GLUT4-mOrange-positive cells being examined in each experiment (Lumeng et al., 2007; Kawaguchi et al., 2008).

### RNA Isolation and Quantitative Real-Time PCR

L6 cells were starved for 2 h in an α-MEM solution, incubated with different inhibitors for specific time, and then treated with either insulin or neferine. Then, total cellular RNA was extracted using TRIzol reagent and dissolved in diethyl pyrocarbonate water. The corresponding cDNA was obtained using a reverse transcription kit. Real-time reaction was performed using the 7,500 rapid real-time PCR system instrument and SYBR dye. The sequences of primers used were: rat GAPDH (NCBI RefSeq NM_017008.4), F: 5’-TACAGCAACAGGGTGGTGGAC-3’, R: 5’- GGGATGGAATTGTGAGGGAGA-3’; rat GLUT4 (NCBI RefSeq NM_012751.1), F: 5’-CTTCCTTCTATTTGCCGTCCTC-3’, and R: 5’-GCTGCTGTTTCCTTCATCCTG-3’.

### Purification of L6 Cell Membrane Fraction

L6 cells were harvested after being starved in an α-MEM solution for 2 h. The L6 cells were then incubated with either 100 nM insulin or 150 μM neferine and then washed with cold PBS and resuspended in HES buffer containing phenyl methane sulfonyl fluoride (PMSF) at 4°C. Then, the cells were lysed by passing through a 22-gauge needle 30 times followed by 30 more passes through a 27-gauge needle. The lysate was then centrifuged at 500 g for 10 min to remove any unbroken cells and then at 18,000 g for 20 min to obtain the membrane fraction. All samples were heated at 65°C for 10 min.

### Western Blotting

Firstly, we treated the cells with insulin and neferine; then, the protein was extracted from L6 cells. The cells were washed with PBS three times and resuspended in HES buffer containing PMSF. Cells were then lysed by passing through a 22-gauge needle 30 times followed by an additional 30 passes through a 27-gauge needle. The lysate was then centrifuged at 500 g for 10 mins, and the supernatant was collected and heated at 65°C for 10 min in sodium dodecyl sulfate (SDS) protein loading buffer. All samples were subjected to 8 or 10% SDS-polyacrylamide gel electrophoresis and then transferred to a polyvinylidene fluoride (PVDF) membrane. The PVDF membrane was later incubated with primary and HRP-conjugated secondary antibodies. Protein bands were incubated using ECL kits and then detected and quantified using the Chemi‐Doc XRS system (Bio-Rad, Hercules, CA).

### Data Analysis

To determine whether there were significant differences between groups, we performed Student’s *t*-tests by using Origin 9.0 software (OriginLab, Northampton, MA). Data are shown as the mean ± standard error of the mean. The *n* values represent the number of cells. Differences with *p* < 0.05 were considered statistically significant.

## Results

### Neferine Stimulated Glucose Uptake, Upregulated GLUT4 Expression, and Increased Intracellular Ca^2+^ Concentration in L6 Cells

**Figure 2:**
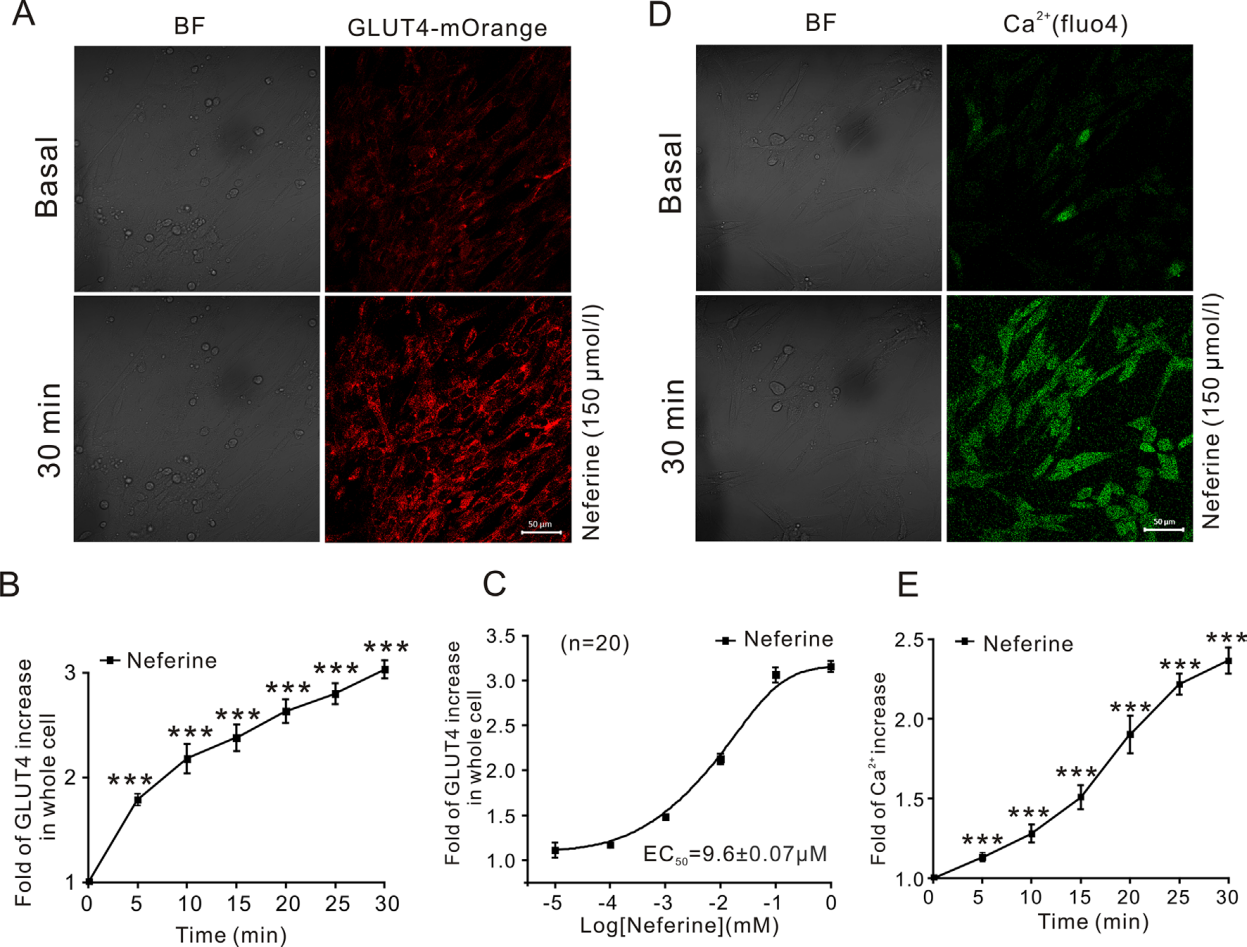
Neferine upregulates GLUT4 expression and intracellular Ca^2+^. **(A)** Images of 150 μM neferine stimulating GLUT4 expression in L6 cells. Scale bar = 50 μm. **(B)** Calculation of fluorescence intensity in myc-GLUT4-mOrange L6 cells using Zen 2010 software. **(C)** Neferine-induced GLUT4 expression occurred in a dose-dependent manner. **(D)** Images of 150 μM neferine increasing the intracellular Ca^2+^ concentration in L6 cells. Scale bar = 50 μm. **(E)** Calculation of fluorescence intensity in L6 cells using Zen 2010 software. Data represent the fold increase in neferine-induced fluorescence between 0 and 30 min. ***P < 0.001.

In this study, we first investigated the effect of neferine on glucose uptake in L6 cells using the 2-[*N*-(7-nitrobenz-2-oxa-1,3-diaxol-4-yl)amino]-2-deoxyglucose (2-NBDG) assay. As shown in **Figures 1B–D**, neferine increased glucose uptake in both insulin-resistant (IR) and non-insulin-resistant L6 cells, while 100 nM insulin induced an increase only in non-insulin-resistant cells. Additionally, neferine induced glucose uptake in a dose-dependent manner. To further investigate whether neferine promoted glucose uptake by increasing the expression of GLUT4 in L6 cells, we observed changes in cytoplasm GLUT4 levels using a laser confocal microscope. The results indicated that within 30 min of treatment with 150 μM neferine, the red fluorescence protein level (GLUT4-mOrange) in cells rapidly increased by approximately 2.8-fold (**Figures 2A, B**). This response occurred in a dose-dependent manner (**Figure 2C**). Fluorescence of Ca^2+^ labelled with Fluo-4 AM also increased by approximately 2.5-fold. (**Figures 2D, E**).

### Neferine Induced Increases in GLUT4 mRNA and Protein Expression Levels

**Figure 3:**
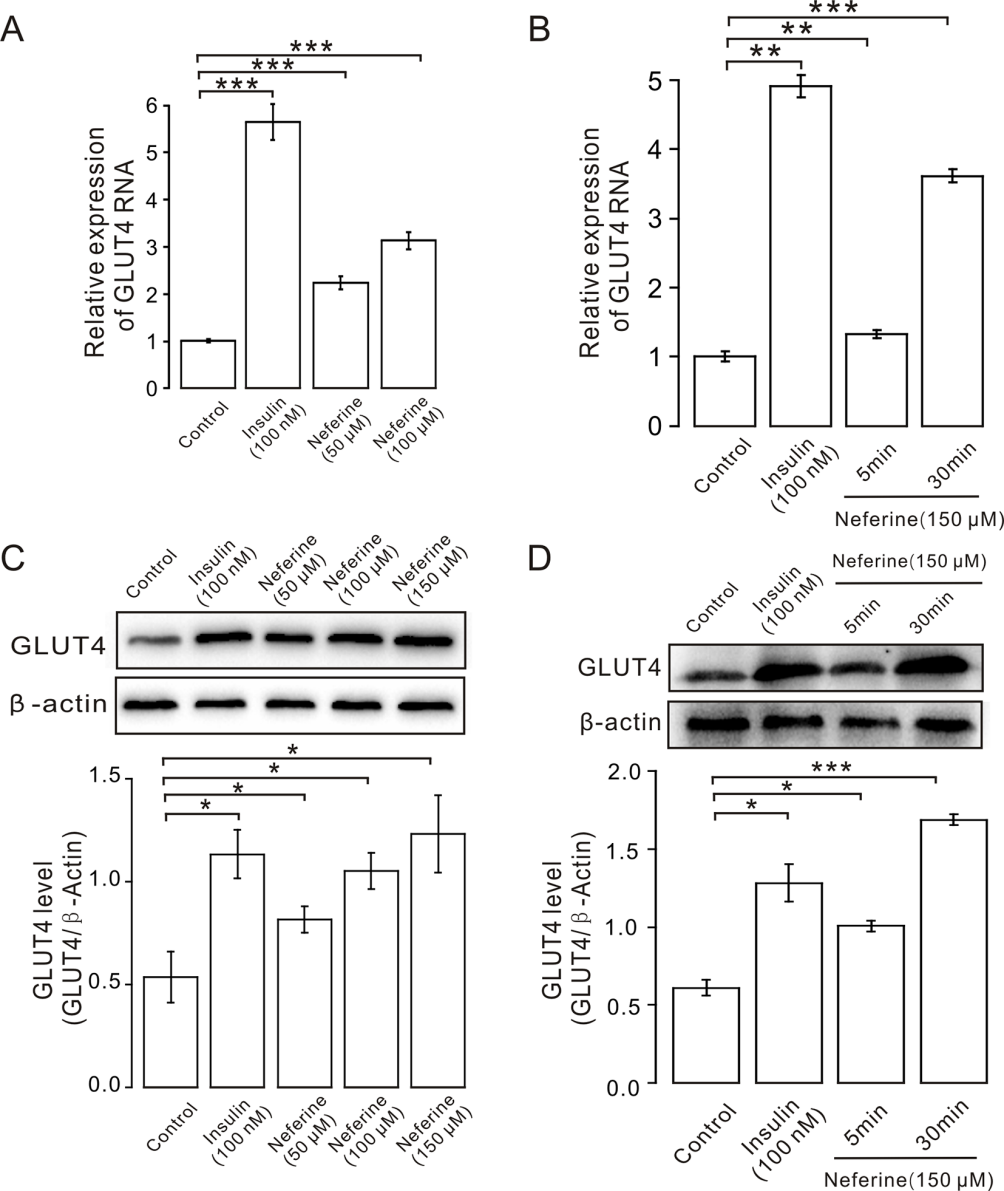
Neferine enhances GLUT4 mRNA and protein expression in L6 cells. **(A)** GLUT4 mRNA expression in L6 cells detected using real-time PCR shows significantly increased mRNA expression in L6 cells in 100 nM insulin, as well as 50 μM, and 100 μM neferine. **(B)** GLUT4 mRNA expression in L6 cells 5 and 30 min after treatment with 150 μM neferine. **(C)** Western blot analysis of GLUT4 protein expression; neferine-induced GLUT4 expression is dose-dependent. **(D)** Effects of 150 μM neferine on GLUT4 protein expression 5 and 30 min after treatment. *P < 0.05; **P < 0.01; ***P < 0.001.

Next, we verified the GLUT4 mRNA and protein expression levels in L6 cells. The results revealed that, following the addition of 50, 100, or 150 μM neferine in 30 min, GLUT4 protein expression increased by approximately 1.5-fold, 2.0-fold, and 2.3-fold respectively, compared to the controls. As positive control, insulin-induced cellular GLUT4 expression increased by approximately 2.1-fold (**Figure 3C**). GLUT4 mRNA expression in L6 cells treated with 100 nM insulin increased by approximately 5.5-fold; the mRNA expression level of GLUT4 induced by neferine at concentrations of 50 and 100 μM increased by approximately 2.2-fold and three-fold, respectively (**Figure 3A**). Next, we evaluated GLUT4 mRNA expression after 5- and 30-min treatment with 150 μM neferine and found an increase of approximately 1.3-fold and 3.5-fold (**Figure 3B**); the protein expression of GLUT4 induced by 150 μM neferine after 5- and 30-min treatment increased by approximately 1.4-fold and 2.7-fold, respectively (**Figure 3D**). This demonstrated that neferine promotes GLUT4 expression in L6 cells.

### Neferine Promoted GLUT4 Fusion With The Plasma Membrane

**Figure 4:**
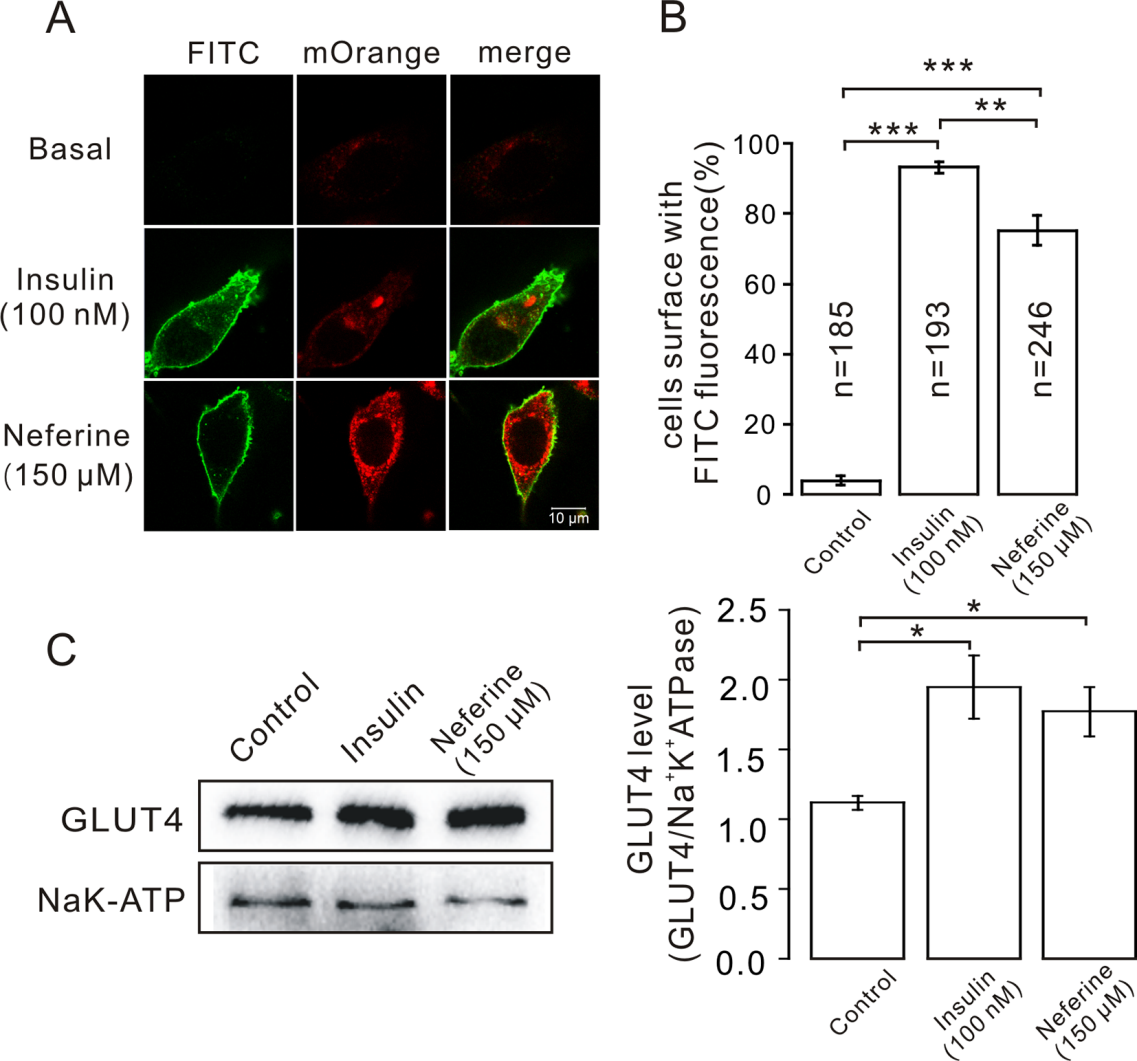
Neferine promotes fusion of GLUT4 with the plasma membrane in L6 cells. **(A)** FITC fluorescence assay in myc-GLUT4-mOrange cells treated with 150 μM neferine or 100 nM insulin. Scale bar: 10 μm. **(B)** Number and quantitation of FITC green fluorescence cells. **(C)** Treatment with 100 nM insulin or 150 μM neferine for 30 min significantly increases GLUT4 expression levels on the plasma membrane. *P < 0.05; **P < 0.01; ***P < 0.001.

After neferine-induced GLUT4 expression was observed in L6 cells using confocal laser scanning microscopy, we considered whether neferine could also facilitate the fusion of GLUT4 with the plasma membrane. So, to confirm this, we first transfected GV348-myc-GLUT4-mOrange lentivirus into L6 cells to perform immunofluorescence experiments. The red fluorescence of GLUT4-mOrange and the green fluorescence of FITC-myc were then detected using laser confocal microscopy. As a result, we found that neferine and insulin can both promote GLUT4 fusion with plasma membrane (**Figure 4A**). Quantification of this effect revealed that the percentage of FITC positive cells were 90 and 75%, respectively (**Figure 4B**). Next, we extracted the membrane protein and detected the changes in GLUT4 expression levels. We found that compared with the control, GLUT4 protein in the membrane protein was significantly increased after treatment with 100 nM insulin and 150 μM neferine (**Figure 4C**). So, we concluded that neferine does indeed promote GLUT4 fusion with the plasma membrane in L6 cells.

### Increased GLUT4 Expression Induced by Neferine Is Mediated by the PKC and AMPK Pathways

**Figure 5:**
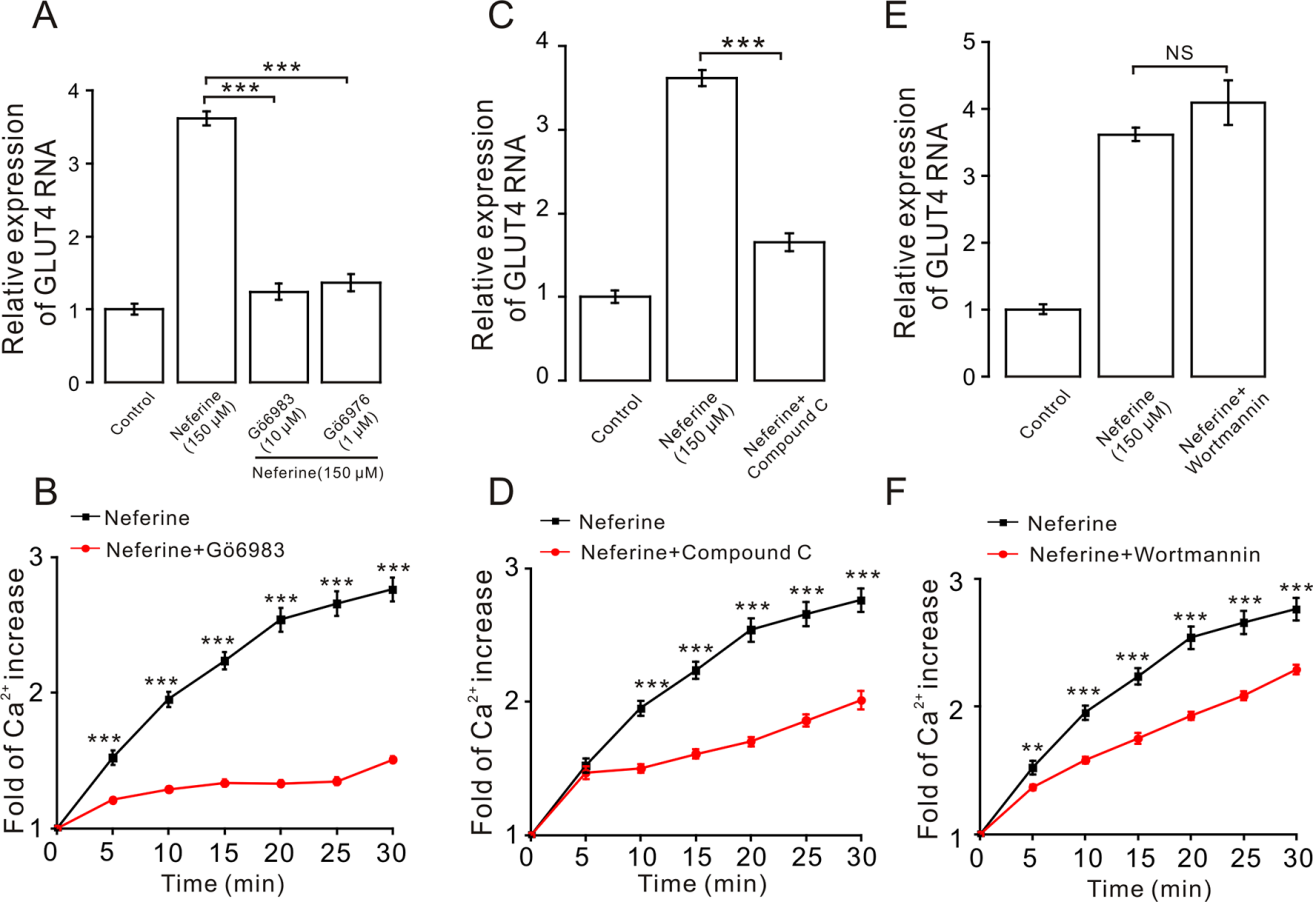
Expression of neferine-induced GLUT4 is mediated by the PKC and AMPK signaling pathways. **(A)** GÖ6983 (10 µM) and GÖ6976 (1 µM) strongly inhibit neferine-increased GLUT4 mRNA expression. **(B)** GÖ6983 inhibits Ca^2+^ concentration enhanced by neferine in L6 cells. **(C)** AMPK inhibitor compound C (10 µM) also significantly inhibits the increase of neferine-induced GLUT4 expression. **(D)** Effects of compound C (10 µM) on Ca^2+^ concentration induced by 150 µM neferine in L6 cells. **(E)** IP_3_K blocker wortmannin (10 µM) has no effect on the increase of GLUT4 expression in the L6 cells induced by neferine. **(F)** Wortmannin (10 µM) has an inhibitory effect on neferine-induced Ca^2+^ concentration. NS *P* > 0.05; **P < 0.01; ***P < 0.001.

We next investigated the mechanism underlying neferine-induced GLUT4 expression. First, after incubating cells with PKC blockers GÖ6983 (10 µM) and GÖ6976 (1 µM), AMPK blocker compound C (10 µM), or PI3K inhibitor wortmannin (10 µM) for 30 min, cells were treated with neferine for 30 min and changes in GLUT4 expression in L6 cells were observed using real-time PCR. Results showed that GÖ6983, GÖ6976, and compound C inhibited any neferine-induced GLUT4 expression (**Figures 5A, C**), but wortmannin had no significant effect (**Figure 5E**). These results indicated that neferine regulated intracellular GLUT4 expression through the PKC and AMPK pathways, but not through the PI3K pathway. Moreover, all three signaling pathway compounds tested significantly inhibited neferine-induced intracellular Ca^2+^ concentration (**Figures 5B, D, F**). This suggested that Ca^2+^ may be involved in promoting GLUT4 expression, but the specific regulatory mechanism was not clear.

**Figure 6:**
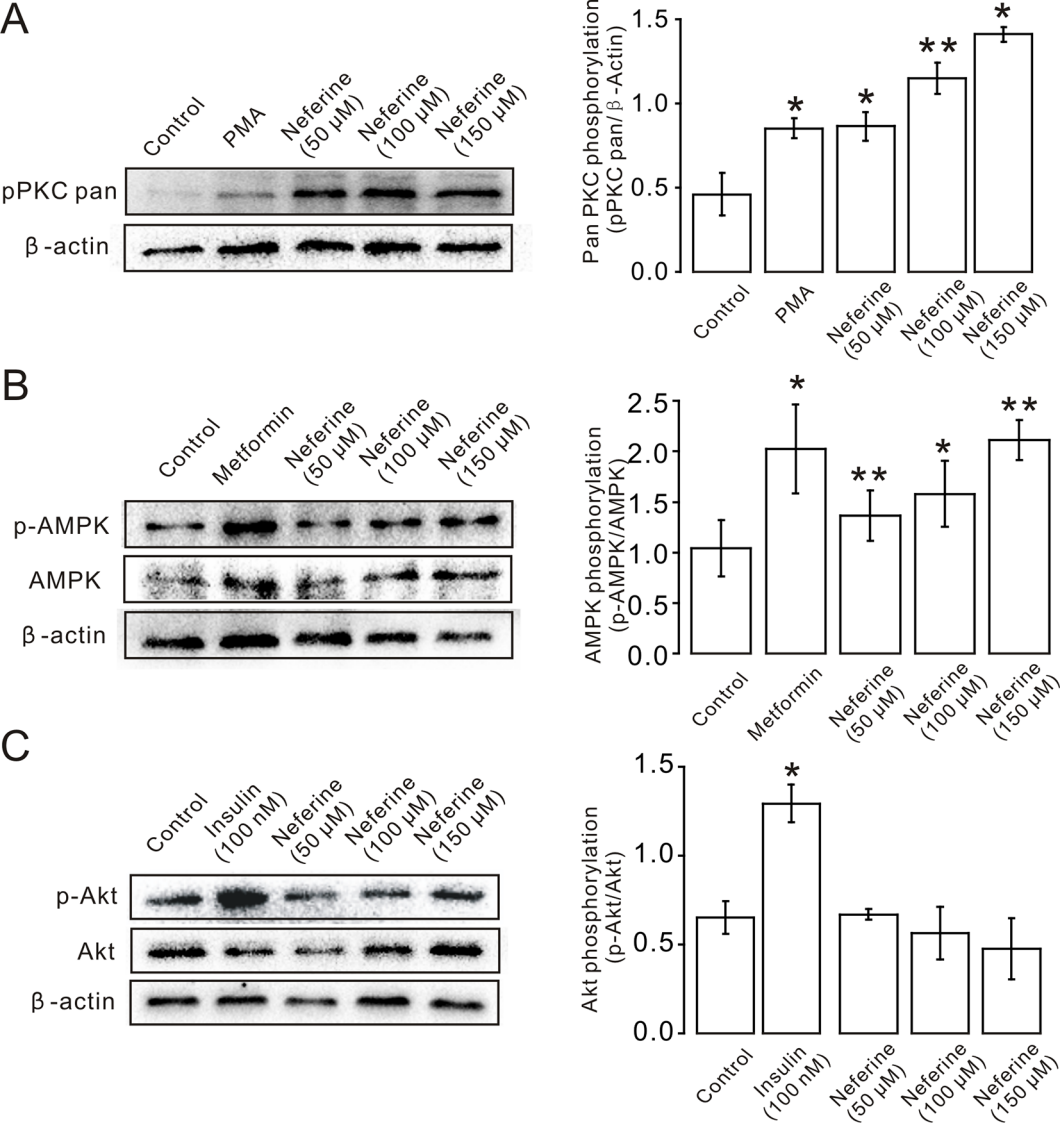
Neferine induces GLUT4 expression in L6 cells by promoting phosphorylation of the PKC and AMPK signaling pathways. **(A**) Neferine induces an increase (∼1.9-fold at 50 µM, ∼2.5-fold at 100 µM, ∼3.0-fold at 150 µM) in PKC phosphorylation levels after 30 min in a dose-dependent manner. 200 nM PMA (positive control) also increases PKC phosphorylation levels (∼1.8-fold). **(B)** Neferine increases AMPK phosphorylation (∼1.3-fold at 50 µM, ∼1.6-fold at 100 µM, ∼2.0-fold at 150 µM) in a dose-dependent manner. 100 μg/ml Metformin (positive control) also increases AMPK phosphorylation (∼1.5-fold). **(C)** Akt phosphorylation is not significantly affected after treatment with 50 µM, 100 µM, or 150 µM neferine. Insulin, however, increases Akt phosphorylation levels. NS *P* > 0.05; *P < 0.05; **P < 0.01.

Next, we verified the protein expression levels in L6 cells. The results indicated that neferine increased phosphorylation of both the PKC and AMPK signaling pathways in a dose-dependent manner. Phosphorylation of PKC and AMPK was significantly increased after stimulation with phorbol 12-myristate 13-acetate (PMA) (Vogt et al., 1991), and using metformin (Lee et al., 2012) as the positive control (**Figures 6A, B**). However, the results showed that there was no significant change in the phosphorylation of Akt after treatment with the three concentrations of neferine previously described (**Figure 6C**). These results confirmed the earlier findings that neferine promoted GLUT4 expression through the PKC and AMPK pathways.

### The G protein-PLC Signal Pathway Is Involved in Neferine-Induced GLUT4 Expression in L6 Cells

**Figure 7:**
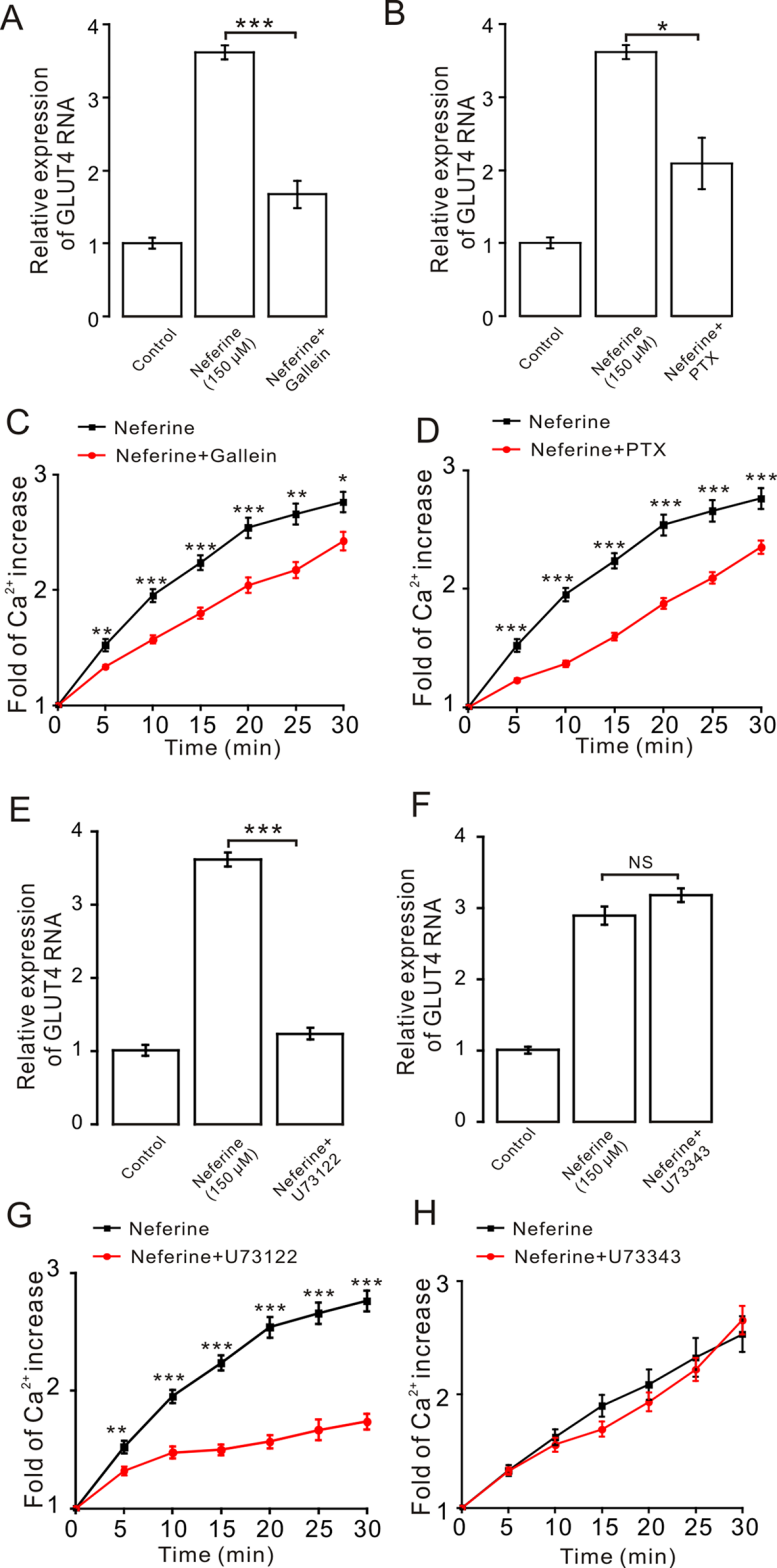
The G protein-PLC signal pathway is involved in neferine-induced GLUT4 expression in L6 cells. **(A)** G protein inhibitor gallein (100 µM) significantly inhibits neferine-induced GLUT4 mRNA expression. **(B)** Treatment with G protein inhibitor PTX (100 µM) significantly inhibits neferine-induced GLUT4 mRNA expression. **(C)** Ca^2+^ concentration induced by neferine was inhibited by G protein inhibitor gallein. **(D)** Ca^2+^ concentration induced by neferine was inhibited by G protein inhibitor PTX. **(E)** Significant inhibitory effect of the PLC inhibitor U73122 (1 µM) on neferine-induced GLUT4 mRNA expression. **(F)** U73343 inhibitor (inactive analogue of U73122, 10 µM) has no effect on neferine-induced GLUT4 mRNA expression. **(G)** U73122 inhibits the increase in Ca^2+^ concentration. **(H)** U73343 didn’t inhibit the increase in Ca^2+^ concentration. NS *P* > 0.05; *P < 0.05; **P < 0.01; ***P < 0.001.

As the aforementioned experiment indicated, neferine promotes cellular GLUT4 expression *via* the AMPK and PKC signaling pathways (**Figures 5** and **6**). For our next experiment, we incubated L6 cells for 6–8 h with G_α_ protein inhibitor PTX (100 µM) or G_β/γ_ protein inhibitor gallein (100 µM). From the results, we determined that both PTX and gallein inhibited neferine-induced GLUT4 mRNA expression (**Figures 7A, B**) as well as intracellular Ca^2+^ concentration (**Figures 7C, D**). Subsequently, we also cultured L6 cells for 30 min with the PLC inhibitor U73122 (1 µM) or U73343 (inactive analogue of U73122, 10 µM) and found that U73122 significantly inhibited neferine-induced GLUT4 expression, but U73343 had no significant effect (**Figures 7E, F**). Additionally, only U73122 had significant inhibitory effects on neferine-induced Ca^2+^ concentration; U73343 was without effect on it (**Figures 7G, H**).

**Figure 8:**
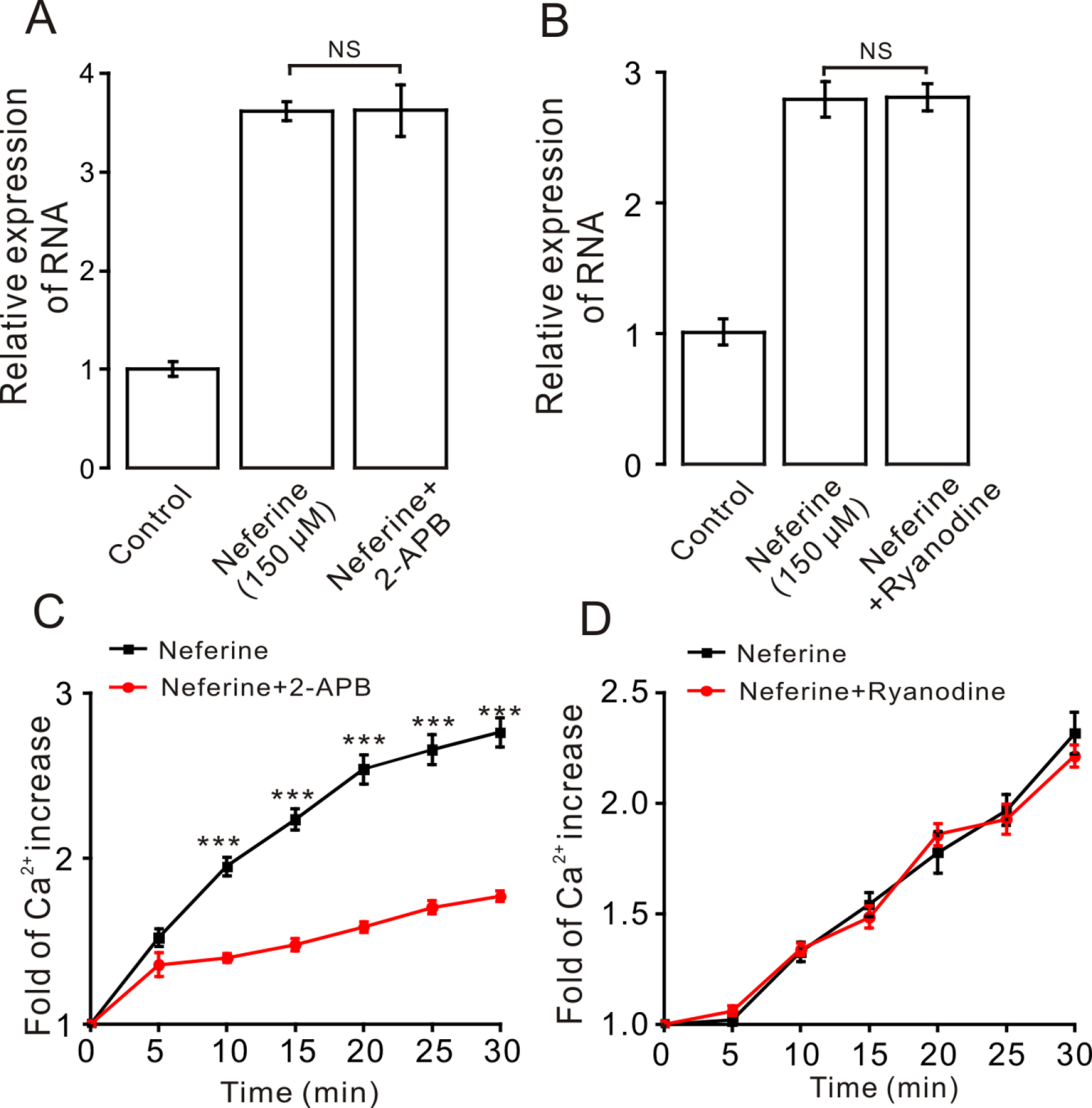
IP3R mediates neferine-triggered intracellular Ca^2+^ release. **(A)** Incubation of IP3R receptor blocker 2-APB (100 µM) for 30 min has no effect on neferine-induced GLUT4 mRNA expression. **(B)** Incubation of ryanodine (RyR blocker, 30 µM) for 3 h has no effect on neferine-enhanced GLUT4 expression. **(C)** 2-APB inhibits the increase in Ca^2+^ concentration. **(D)** Neferine-stimulated intracellular Ca^2+^ concentration was not inhibited by Ryanodine. NS *P* > 0.05; ***P < 0.001.

Moreover, to certify if intracellular calcium channels involve in the process of neferine-stimulated GLUT4 expression and Ca^2+^ concentration, we used 100 μM 2-APB to block IP3R-regulated intracellular Ca^2+^ release. The result revealed that incubation of 2-APB for 30 min had no effect on neferine-mediated GLUT4 translocation under 2 mM extracellular Ca^2+^, but it evidently inhibited neferine-triggered Ca^2+^ release (**Figures 8A, C**). The ryanodine receptor (RyR), another channel that releases Ca^2+^ in the sarcoplasmic reticulum (SR)/endoplasmic reticulum (ER), has attracted much attention due to its manipulation of intracellular Ca^2+^ output (Zeng et al., 2014). Incubation of 30 μM ryanodine (RyR inhibitor) for 3 h had no effect on either GLUT4 translocation or intracellular Ca^2+^ increase mediated by neferine (**Figures 8B, D**). Thus, the G protein-PLC signal pathway is involved in neferine-induced GLUT4 expression in L6 cells.

### Effects of Ca^2+^ in Neferine-Induced GLUT4 Expression, Glucose Uptake, and GLUT4 Fusion With The Plasma Membrane in L6 Cells

**Figure 9:**
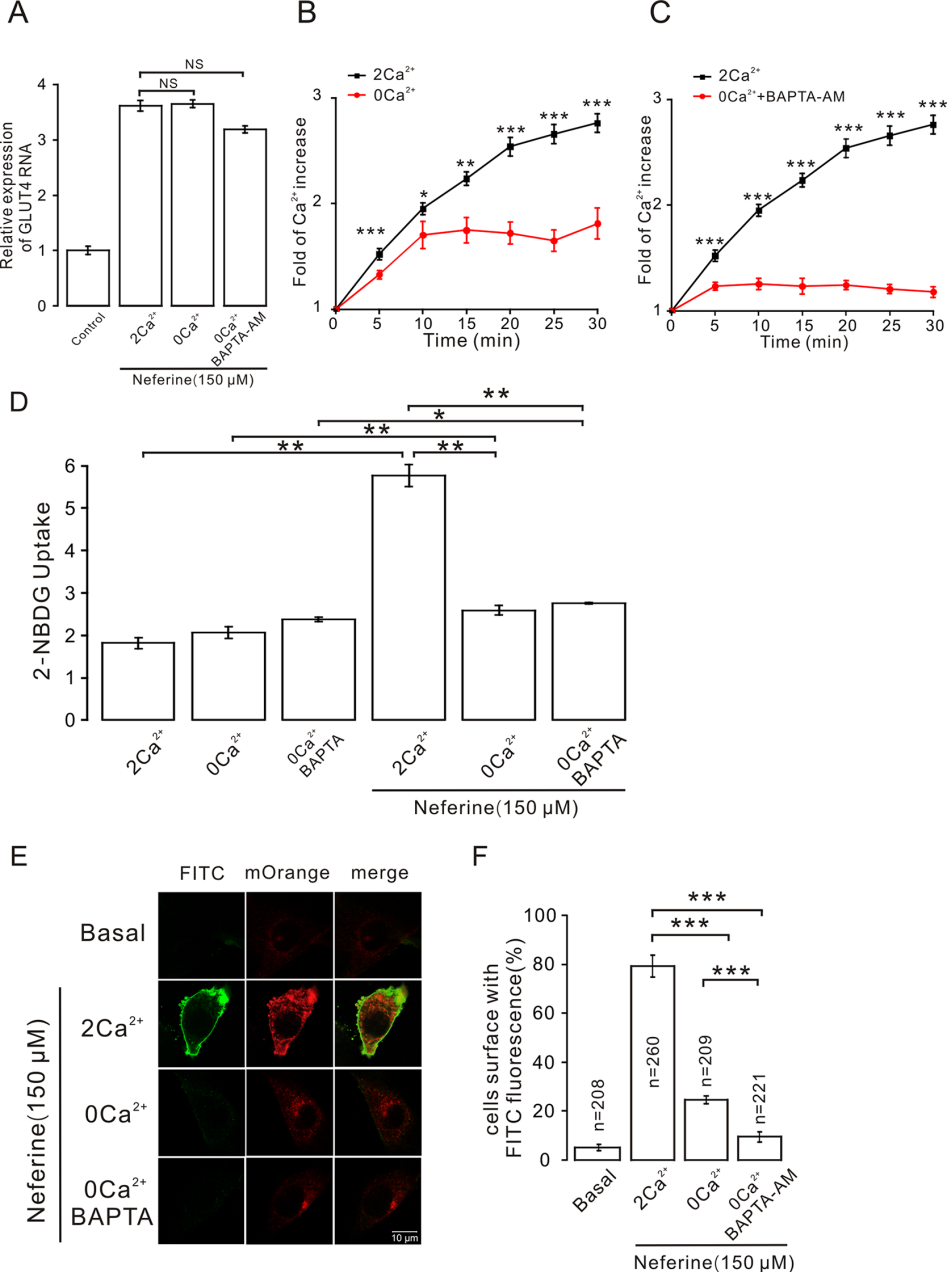
Intracellular calcium chelator BAPTA-AM does not inhibit neferine-induced GLUT4 expression but inhibits GLUT4 fusion with the plasma membrane and glucose uptake enhanced by neferine. **(A)** Treatment with 150 µM neferine in L6 cells under the condition of 0 mM extracellular Ca^2+^, 2 mM extracellular Ca^2+^, and 0 mM extracellular Ca^2+^ + 10 μM BAPTA-AM yields no significant differences in GLUT4 expression. **(B)** Treatment with 150 μM neferine in L6 cells with 0 mM extracellular Ca^2+^ showed significant inhibition of intracellular Ca^2+^ concentration. **(C)** After treatment with 0 mM extracellular Ca^2+^ + 10 μM BAPTA-AM, the increase in intracellular Ca^2+^ concentration by 150 μM neferine was inhibited. **(D)** Treatment with 2 mM extracellular Ca^2+^, 0 mM extracellular Ca^2+^, and 0 mM extracellular Ca^2+^ + 10 μM BAPTA-AM yields no significant differences in intracellular glucose uptake. Treatment with 2 mM extracellular Ca^2+^ after neferine treatment significantly increases glucose uptake in L6 cells. Treatment with 0 mM extracellular Ca^2+^ and 0 mM extracellular Ca^2+^ +10 μM BAPTA-AM and neferine inhibit glucose uptake in L6 cells. **(E)** Treatment of myc-GLUT4-mOrange L6 cells with neferine under the displayed conditions showing laser confocal microscopy detection of GLUT4-mOrange red fluorescence in L6 cells and FITC green fluorescence on cell membranes. Scale bar: 10 μm. **(F)** Activated FITC fluorescence cell count and quantitation in L6 cells. NS *P* > 0.05; *P < 0.05; **P < 0.01; ***P < 0.001.

As described previously, we found that neferine promotes the increase in intracellular Ca^2+^ concentration, and after treatment with inhibitors of different signaling pathways, the increase in Ca^2+^ concentration was significantly inhibited. This suggested that Ca^2+^ plays an important role in neferine-induced GLUT4 expression and glucose uptake in L6 cells. To test this speculation, we first treated cells with neferine and added either 2 mM extracellular Ca^2+^, 0 mM extracellular Ca^2+^, or 0 mM extracellular Ca^2+^ + 10 μM BAPTA-AM (a Ca^2+^ chelator). We found that the increase in GLUT4 expression was mostly unaffected in each case (**Figure 9A**). In addition, the increase in intracellular Ca^2+^ concentration as induced by 150 μM neferine was partially inhibited under the condition of 0 mM extracellular Ca^2+^ and completely inhibited under the condition of 0 mM extracellular Ca^2+^ + 10 μM BAPTA-AM (**Figures 9B, C**). These results indicated that Ca^2+^ does not directly regulate neferine-induced GLUT4 expression in L6 cells.

However, we used a 2-NBDG assay of cultured cells with and without added neferine in 2 h under conditions of 2 mM extracellular Ca^2+^, 0 mM extracellular Ca^2+^, or 0 mM extracellular Ca^2+^ + 10 μM BAPTA-AM. In the absence of neferine, there was no significant difference in glucose uptake. Under the condition of cells treated with neferine and 2 mM extracellular Ca^2+^, neferine-induced glucose uptake increased by nearly three-fold. Under the condition of treatment with neferine and 0 mM extracellular Ca^2+^, neferine-induced glucose uptake was significantly lower than the 2 mM extracellular Ca^2+^ condition. Also, under the condition of treatment with neferine and 0 mM extracellular Ca^2+^ + 10 μM BAPTA-AM, neferine-induced glucose uptake was significantly inhibited (**Figure 9D**). These results suggested that Ca^2+^ plays an important role in neferine-induced glucose uptake in L6 cells.

Next, we performed immunofluorescence experiments to detect changes in FITC green fluorescence in myc-GLUT4-mOrange L6 cells under the conditions of 2 mM extracellular Ca^2+^, 0 mM extracellular Ca^2+^, or 0 mM extracellular Ca^2+^ + 10 μM BAPTA-AM. In basal cells, FITC green fluorescence of the cell membrane was almost not detected in L6 cells transfected with myc-GLUT4-mOrange. When 150 μM neferine was added to stimulate cells, however, we observed a significant increase in FITC green fluorescence on the cell membrane surface. Interestingly, under the conditions of 0 mM extracellular Ca^2+^ and 0 mM extracellular Ca^2+^ +10 μM BAPTA-AM, FITC green fluorescence was almost undetectable on the cell membrane (**Figures 9E, F**). Therefore, the neferine-induced fusion of GLUT4 with the cell membrane is indeed regulated by the Ca^2+^ signal.

## Discussion

Neferine is a dibenzyl quinoline alkaloid extracted from lotus seeds and is known to have many types of biological activity. *In vitro* experiments from other studies have shown that neferine can reduce the collagen production of myocardial fibroblasts induced by high glucose levels in diabetic mice, and that it also has an anti-fibrotic effect (Liu et al., 2016). Moreover, neferine can also inhibit hyperglycemia-induced endothelial dysfunction and apoptosis, providing a basis for the treatment of vascular complications of diabetes (Guan et al., 2014). In this present study, our findings showed that neferine upregulated GLUT4 expression through the G protein-PLC-PKC and AMPK pathways, and that it also promoted GLUT4 fusion with the plasma membrane and glucose uptake. Also, neferine increased intracellular Ca^2+^ through the G protein-PLC-IP_3_-IP_3_R pathway, and neferine-induced GLUT4 fusion with the plasma membrane and glucose uptake are both Ca^2+^-dependent in L6 cells.

GLUT4 is a 12-transmembrane facilitative glucose transporter that is primarily expressed in muscle and adipose tissues. Genetic ablation of the GLUT4 gene, specifically in mouse muscle or adipose tissue, results in impaired glucose uptake, hyperinsulinemia, and peripheral insulin resistance (Abel et al., 2001; Leney and Tavare, 2009). Our previous studies have reported that neferine can promote GLUT4 expression in L6 cells; however, the mechanism was unclear (Yang et al., 2017). In the present study, using a 2-NBDG assay, we found that neferine promotes glucose uptake in both insulin-resistant (IR) and non-insulin-resistant L6 cells (**Figures 1B, D**). The increase in neferine-induced GLUT4 expression was detected using laser confocal microscopy (**Figures 2A, B**), and protein expression levels as well as mRNA levels also showed distinct increases (**Figure 3**). Neferine also promoted GLUT4 fusion with the plasma membrane (**Figures 4A, B**), and GLUT4 protein levels on the plasma membrane were significantly increased in the presence of neferine (**Figure 4C**).

We then sought to determine the signal transduction pathway involved in the stimulation of GLUT4 expression. Previous studies have reported that PKC participates in insulin-regulated trafficking of the glucose transporter GLUT4 in 3T3L1-GLUT4myc adipocytes (Tsuchiya et al., 2013). Others showed that PKBα/Akt1 plays a significant role in the stimulation of glucose transport by insulin in muscle cells (Wang et al., 1999). It has also been reported that the serine-threonine kinase AMPK mediates glucose transport and translocation of the cardiomyocyte glucose transporter GLUT4 to the cell surface through activation of the nitric oxide-signaling pathway in isolated heart muscles (Li et al., 2004). Together, these studies suggested that the PKC, PKB, and AMPK pathways are all involved in GLUT4 translocation. From our research, we found that neferine promoted GLUT4 expression through the PKC and AMPK pathways, but not through the PKB pathway (**Figures 5** and **6**). Others have also shown that, in HepG2 hepatocellular carcinoma cells, neferine-induced apoptosis is associated with increased intracellular Ca^2+^ (Poornima et al., 2013). Our results also confirmed that neferine-promoted expression of GLUT4 in L6 cells is associated with a significant increase in intracellular Ca^2+^ (**Figures 2D, E**). So, we speculated that Ca^2+^ may be involved in the process of neferine promoting GLUT4 expression.

In skeletal muscle and cardiac cells, increased intracellular Ca^2+^ concentration may be caused by the entry of extracellular Ca^2+^ or by the insulin-induced release of SR Ca^2+^, which is released through IP_3_ connecting channels (Whitehead et al., 2001; Contreras-Ferrat et al., 2010; Contreras-Ferrat et al., 2014a). In addition, consistent with the previous results in skeletal muscle cells, the transient IP_3_R-dependent state of Ca^2+^ was found to contribute to the increase in insulin-dependent glucose uptake through GLUT4 vesicular translocation to the cell membrane surface (Contreras-Ferrat et al., 2014b). Therefore, in our study, we then sought to clarify how exactly Ca^2+^ regulates neferine-induced GLUT4 expression in L6 cells. Treatment with the G_β/γ_ protein blocker gallein and G_α_ protein blocker PTX were both shown to significantly block increased neferine-induced GLUT4 expression and intracellular Ca^2+^ concentration (**Figure 7**). The PLC blocker U73122 also inhibited neferine-induced GLUT4 expression and Ca^2+^ concentration in L6 cells. An inactive analogue of U-73122, U-73343, was without effect on either GLUT4 expression or Ca^2+^ concentration increase (**Figure 7**). However, downstream of PLC is IP_3_ and its inhibitor 2-APB had no effect on GLUT4 expression, but it did significantly block intracellular Ca^2+^ (**Figures 8A, C**). Additionally, ryanodine inhibitor did not have any impact on neferine-triggered Ca^2+^ elevation or GLUT4 expression (**Figures 8B, D**). This indicated that neferine promotes GLUT4 expression *via* the G protein-PLC-PKC pathway and induces the increase in intracellular Ca^2+^ concentration through the G protien-PLC-IP_3_-IP_3_R pathway.

Other studies have proven that the activation of AMPK and increases in cytosolic Ca^2+^ are responsible for mediating the increase in muscle contraction–induced glucose transport activity (Wright et al., 2004). BAPTA-AM, as an intracellular Ca^2+^chelator, inhibits the increase in Ca^2+^ concentration (Wright, 2007; Li et al., 2014). When we treated cells with neferine under the conditions of 0 mM extracellular Ca^2+^ or 0 mM extracellular Ca^2+^ + 10 μM BAPTA-AM, we found that Ca^2+^ concentration was partially inhibited under the condition of 0 mM extracellular Ca^2+^ and almost completely inhibited under the condition of 0 mM Ca^2+^ + 10 μM BAPTA-AM. At the same time, the increase in GLUT4 expression was not affected (**Figure 9A**). However, in subsequent experiments, we found that under the condition of 0 mM extracellular Ca^2+^ + 10 μM BAPTA-AM, the neferine-induced increase of glucose uptake was significantly inhibited (**Figure 9D**), indicating that Ca^2+^ was indeed involved in the process of promoting glucose uptake by neferine.

These findings left us with the question of how Ca^2+^ regulates neferine to promote glucose uptake. Some studies have reported that the GLUT4 trafficking process consists of six steps including endocytosis, degradation, sorting, sequestration, release, and tethering/docking/fusion (Brewer et al., 2014). Although Ca^2+^ did not affect neferine-induced GLUT4 expression in our study (**Figure 9A**), it is likely that Ca^2+^ may be involved in the process of neferine promoting GLUT4 fusion with the cell membrane. Immunofluorescence results showed that, under the condition of 0 mM extracellular Ca^2+^ + 10 μM BAPTA-AM, neferine-induced GLUT4 fusion with the plasma membrane was completely blocked (**Figures 9E, F**). This means that neferine promotes the binding of GLUT4 vesicles to the plasma membrane by promoting an increase in intracellular Ca^2+^ concentration, and thus increasing glucose uptake.

**Figure 10:**
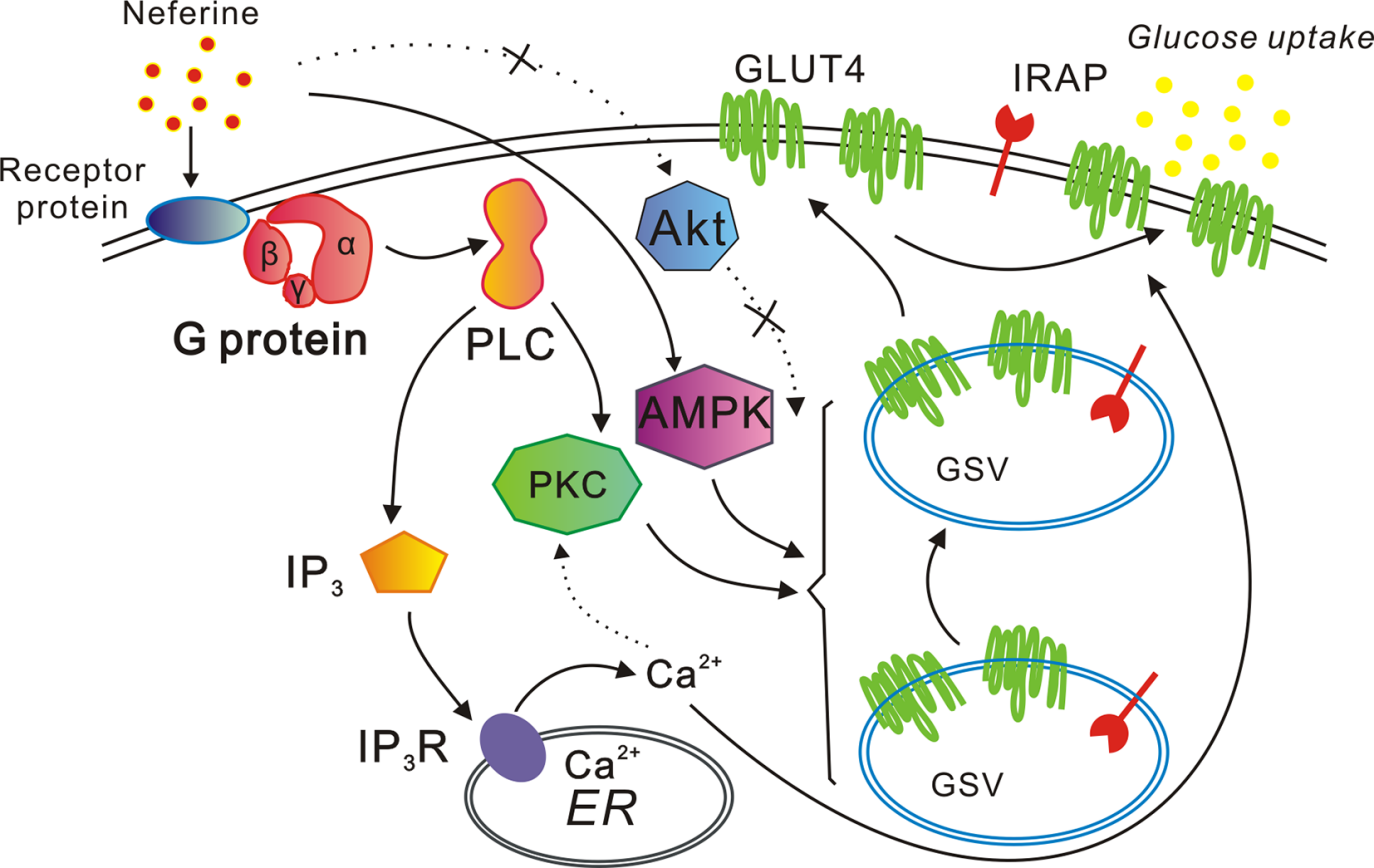
Proposed model of the neferine-induced increase in glucose uptake. Neferine promotes GLUT4 expression *via* the G protein-PLC-PKC and AMPK pathways, thereby enhancing both GLUT4 fusion with the plasma membrane and glucose uptake. Neferine also increases intracellular Ca^2+^ concentration through the G protein-PLC-IP_3_-IP_3_R pathway in L6 cells. Thus, neferine-induced GLUT4 fusion with the plasma membrane and glucose uptake are both Ca^2+^-dependent.

In conclusion, our study indicated that neferine promotes GLUT4 expression *via* the G protein-PLC-PKC and AMPK pathways, thus promoting both GLUT4 fusion with the plasma membrane and glucose uptake in L6 cells. Neferine also increases intracellular Ca^2+^ concentration through the G protein-PLC-IP_3_-IP_3_R signaling pathway (**Figure 10**). Lastly, neferine-induced GLUT4 fusion with the PM and glucose uptake are Ca^2+^-dependent. Besides, previous studies on pharmacokinetics of neferine have shown that neferine has a good utilization rate in animals (Zhao et al., 2007). Based on these findings, our study further explained the molecular mechanism of neferine-induced glucose uptake in L6 cells. Therefore, we believe that neferine has the potential to become an effective agent in the treatment of diabetes mellitus. This is promising news for patients with type 2 diabetes.

## Data Availability

All datasets generated for this study are included in the manuscript and the **Supplementary files**.

## Author Contributions

PZ and XY were involved in the study design. PZ and DT conducted the experiments, analyzed the data, and wrote the manuscript. PZ and XY had primary responsibility for the final content. GS, QM and JL performed the data analyses, JS and QHL helped perform the analysis with constructive discussions. All authors have approved the final version of the manuscript and agree to be accountable for all aspects of the work.

## Funding

The present study was financially supported by National Natural Science Foundation of China grants (31070744, 81573561 and 81774000), Fundamental Research Funds for the Central Universities, South-Central University for Nationalities (CZR18003, CZP17060 and CZP17048), Wuhan Applied Basic Research Program of Science and Technology (2017060201010217) and Fund for Key Laboratory Construction of Hubei Province (Grant No. 2018BFC360).

## Conflict of Interest Statement

The authors declare that the research was conducted in the absence of any commercial or financial relationships that could be construed as a potential conflict of interest.

## Abbreviations

GLUT4, glucose transporter 4; AMPKα, adenosine monophosphate activated protein kinase α; p-AMPKα, phosphorylated AMPKα; IRAP, insulin regulation of aminopeptidase; PKB (Akt), protein kinase B; PKC, protein kinase C.
