# Supplementary material for: Neferine Promotes GLUT4 Expression and Fusion With the Plasma Membrane to Induce Glucose Uptake in L6 Cells
Source: Front Pharmacol. 2019 Sep 4;10:999. doi: 10.3389/fphar.2019.00999 (PMC6737894; doi:10.3389/fphar.2019.00999)
Supplement: Supplementary file 1 [file Table_1.docx]

Supplementary Material

**Supplementary Figure 1:** **Comparison of  myoblasts and myotubes in terms of neferine-induced GLUT4 expression and glucose uptake.** There are almost no difference for neferine-induced GLUT4 expression observed by confocal between myoblasts and myotubes. For 2-NBDG uptake, the results revealed that differentiation treatment have no effect on control groups. Whether myoblasts or myotubes, 2-NBDG uptake have significant difference between insulin or neferine and control group. However, compared with myotubes, there are also obvious distinction on insulin treatment groups and neferine treatment groups in myoblasts.


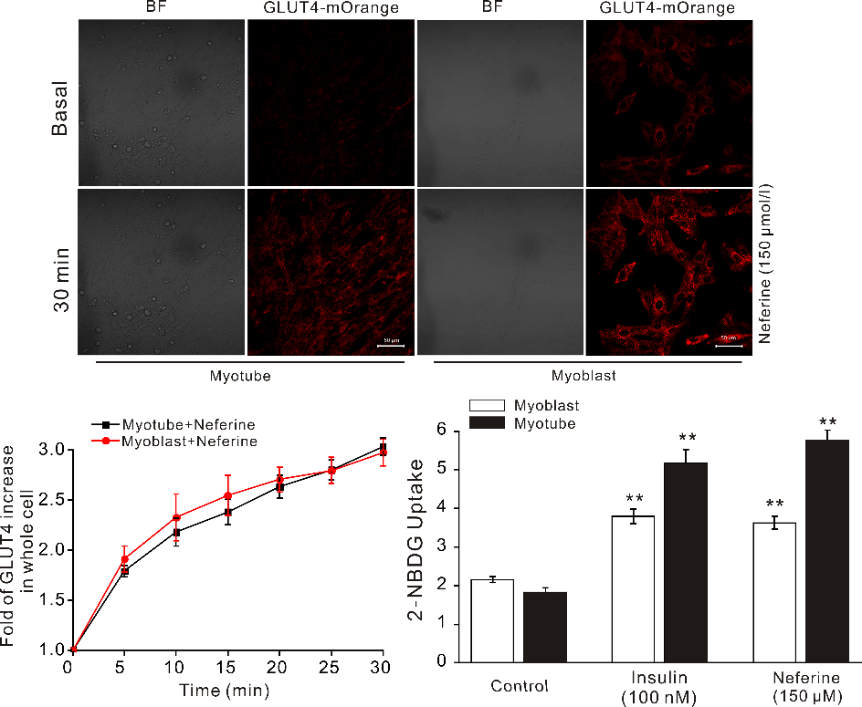


**Fig. S1. Comparison of  myoblasts and myotubes in terms of neferine-induced GLUT4 expression and glucose uptake.**

**Supplementary Figure 2: Effects of differing concentrations of Gӧ6983 (PKC inhibitor) on GLUT4 mRNA expression induced by 150 µM neferine in L6 cells.**

As shown in Fig. 4 A, the increase of GLUT4 expression induced by 150 µM neferine was inhibited by Gӧ6983 (PKC inhibitor). Next, we explored the dose response of Gӧ6983 treatment, and the results indicated that the expression of GLUT4 mRNA was obviously inhibited after treatment with 10 µM, 1 µM, 500 nM, and 50 nM Gӧ6983.


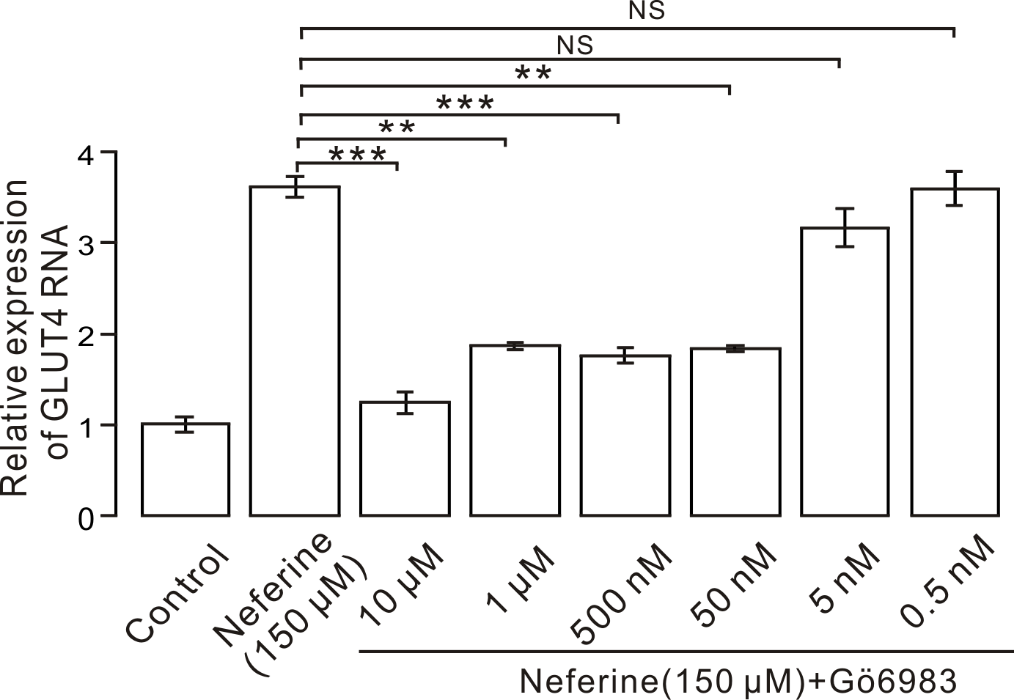


**Fig. S2. Effects of differing concentrations of Gӧ6983 (PKC inhibitor) on GLUT4 mRNA expression induced by 150 µM neferine in L6 cells.**

**Supplementary Figure 3:** **The structure of myc-GLUT4 protein.**

The human c-myc epitope (14 amino acids) was inserted into the first ectodomain of GLUT4. When GLUT4 protein fuse with plasma membrane, c-myc epitope is exposed to outside the cells and antibody could combine with c-myc epitope of GLUT4 protein. Then immunofluorescence method is used to detect membrane-bound GLUT4.


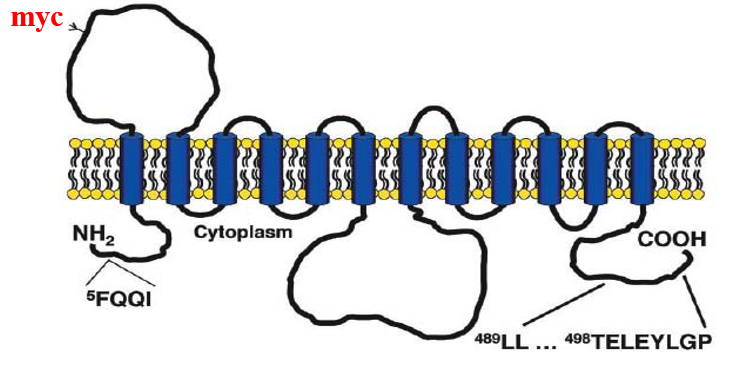


**Fig. S3. The structure of myc-GLUT4 sequence.**

**Supplementary Figure 4: myc-GLUT4-mOrange sequence data.**

The red nucleotides indicated the GLUT4 gene sequence. The human c-myc epitope was inserted into the first ectodomain of GLUT4 as marked by yellow background. The blue nucleotides indicated mOrange protein sequence.


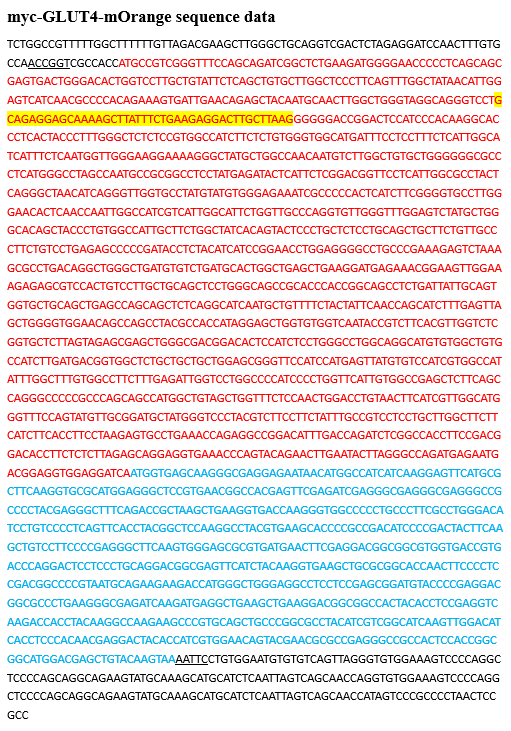


**Supplementary Figure 5: Protein characterizations (amino acids sequence).** The yellow amino acids indicated the human c-myc epitope (14 amino acids) and the blue amino acids indicated mOrange protein amino acid sequence. These amino acids before blue section indicated GLUT4 protein, which have not been tagged.


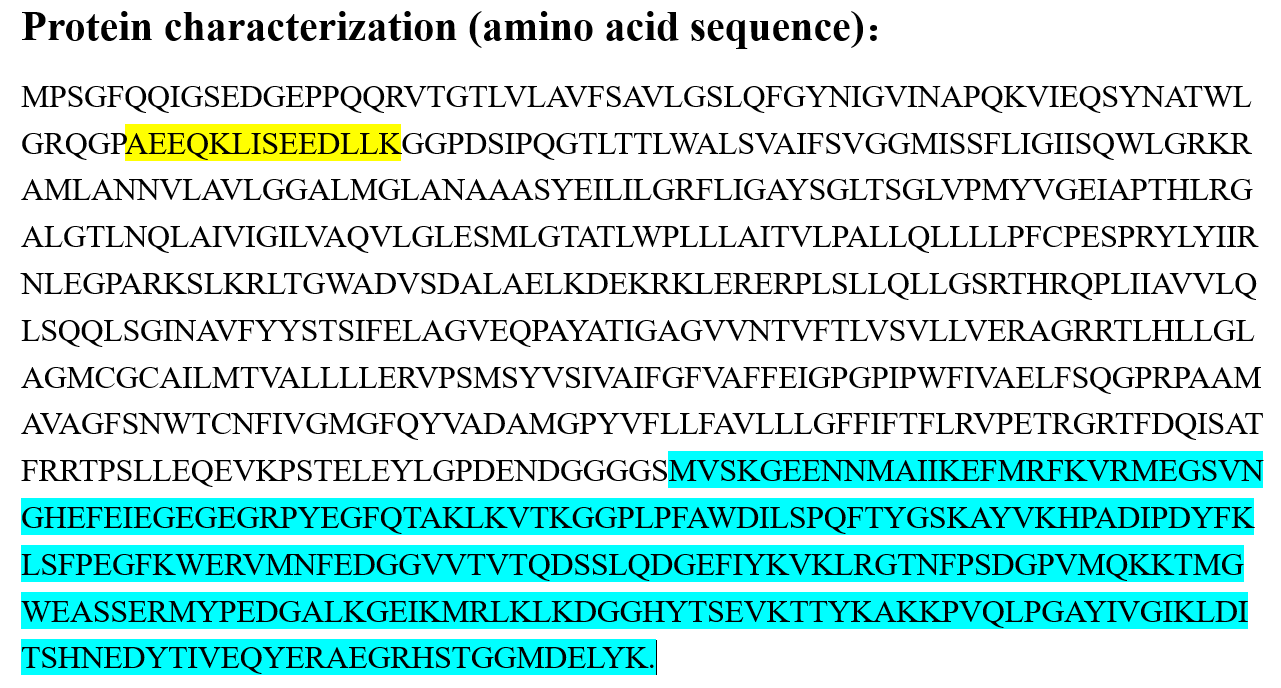


**Supplementary Figure 6: Indinavir effects on neferine-induced glucose uptake in L6 cells.** 100 μM indinavir had a minor inhibitory effect on glucose uptake in unstimulated cells. Insulin-stimulated and neferine-stimulated glucose uptake activity was markedly inhibited by 100 μM indinavir. Neferine-induced stimulation of this activity is dominated by GLUT4.


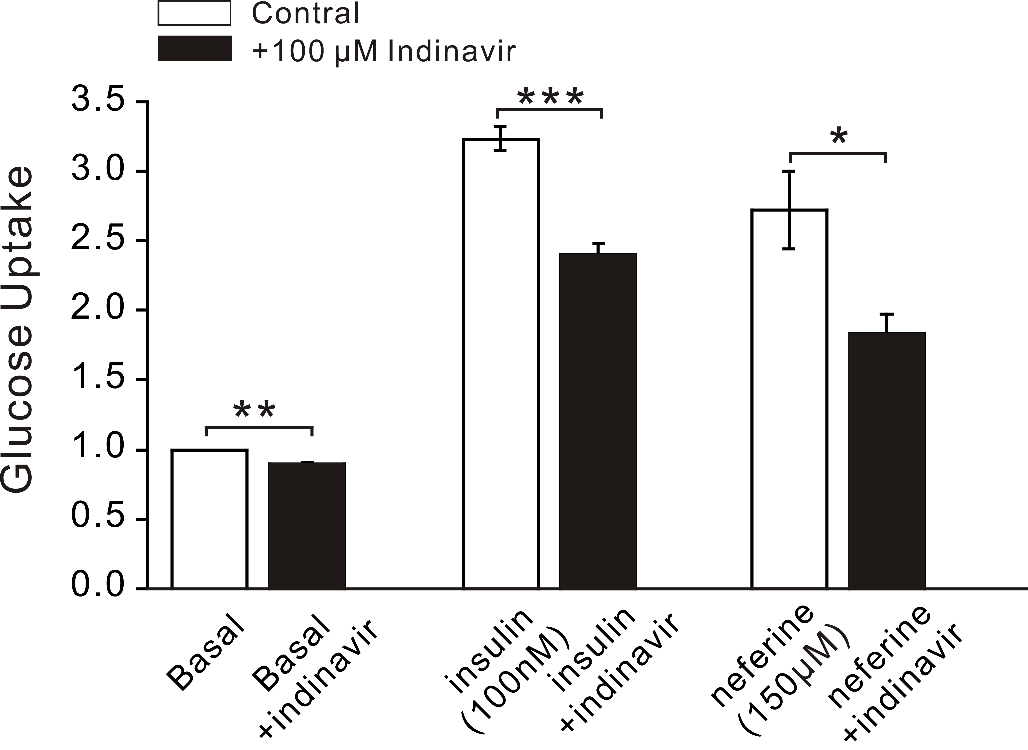


**Fig. S6. Indinavir effects on neferine-induced glucose uptake in L6 cells.**

**Supplementary Figure 7: Cytotoxicity** **and viability assays of L6 cells after neferine treatment.** Neferine treatment have no effect on the cytotoxicity of L6 cells after 30 min or overnight, with no significant difference between the insulin or neferine pretreated groups and the control group. Using MTT colorimetry to detect cell survival, the results showed that the number of cells have hardly change for these three different concentration.


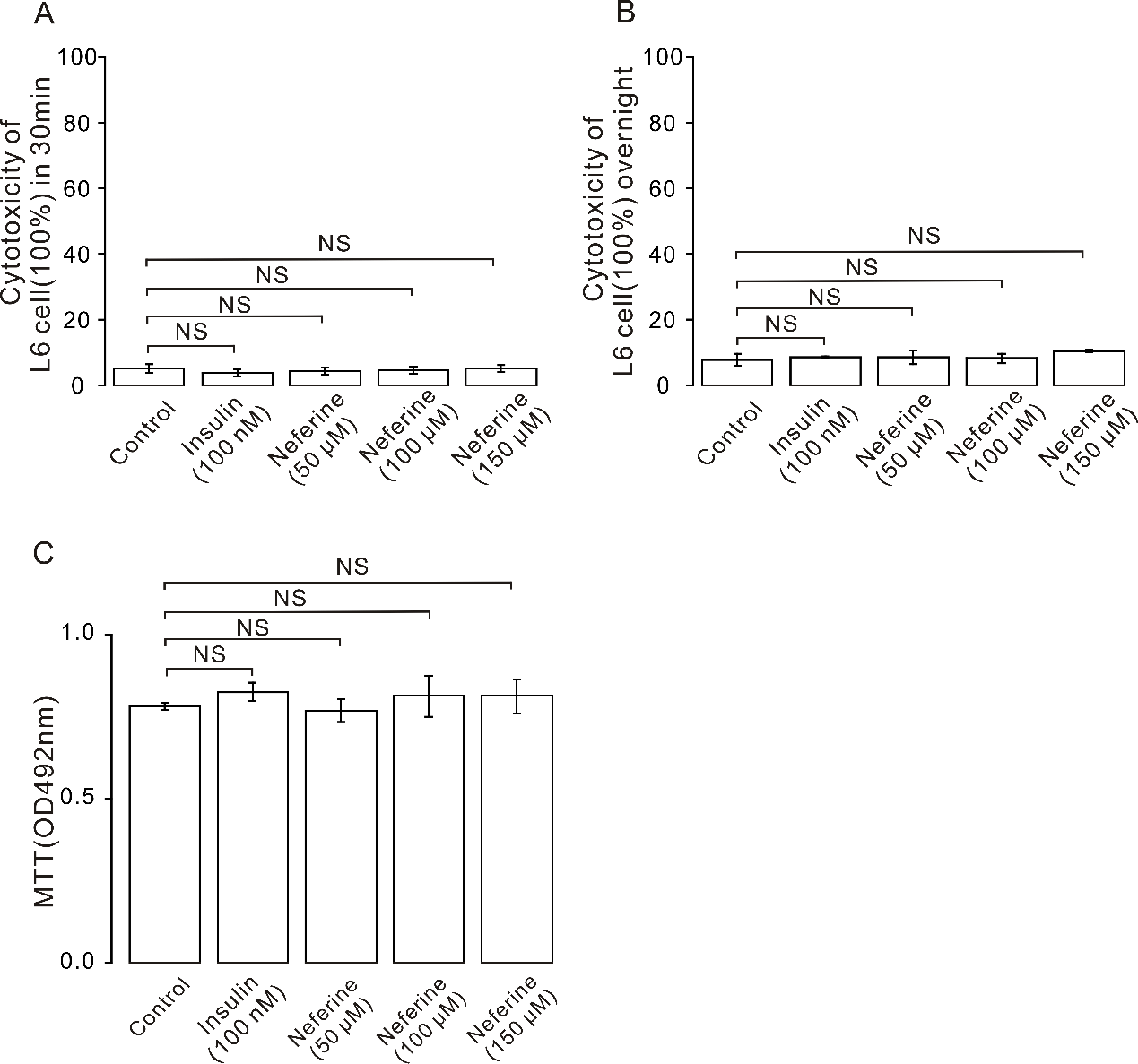


**Fig. S7. Cytotoxicity and viability assays of L6 cells after neferine treatment.**
